# Profiling of Circadian Genes Expressed in the Uterus Endometrial Stromal Cells of Pregnant Rats as Revealed by DNA Microarray Coupled with RNA Interference

**DOI:** 10.3389/fendo.2013.00082

**Published:** 2013-07-08

**Authors:** Hirotaka Tasaki, Lijia Zhao, Keishiro Isayama, Huatao Chen, Nobuhiko Yamauchi, Yasufumi Shigeyoshi, Seiichi Hashimoto, Masa-aki Hattori

**Affiliations:** ^1^Department of Animal and Marine Bioresource Sciences, Graduate School of Agriculture, Kyushu University, Fukuoka, Japan; ^2^Department of Anatomy and Neurobiology, Kinki University School of Medicine, Osaka, Japan; ^3^Graduate School of Medicine, The University of Tokyo, Tokyo, Japan

**Keywords:** uterus endometrial stromal cells, clock genes, clock-controlled genes, DNA microarray, implantation, decidualization, Bmal1 siRNA, Per2-dLuc reporter gene

## Abstract

The peripheral circadian oscillator plays an essential role in synchronizing local physiology to operate in a circadian manner via regulation of the expression of clock-controlled genes. The present study aimed to evaluate the circadian rhythms of clock genes and clock-controlled genes expressed in the rat uterus endometrial stromal cells (UESCs) during the stage of implantation by a DNA microarray. Of 12,252 genes showing significantly expression, 7,235 genes displayed significant alterations. As revealed by the biological pathway analysis using the database for annotation, visualization, and integrated discovery online annotation software, genes were involved in cell cycle, glutathione metabolism, MAPK signaling pathway, fatty acid metabolism, ubiquitin mediated proteolysis, focal adhesion, and PPAR signaling pathway. The clustering of clock genes were mainly divided into four groups: the first group was *Ror*α, *Timeless, Npas2, Bmal1, Id2*, and *Cry2*; the second group *Per1, Per2, Per3, Dec1, Tef*, and *Dbp*; the third group *Bmal2, Cry1, E4bp4, Ror*β, and *Clock*; the fourth group *Rev-erb*α. Eleven implantation-related genes and 24 placenta formation-related genes displayed significant alterations, suggesting that these genes involved in implantation and placenta formation are controlled under circadian clock. Some candidates as clock-controlled genes were evaluated by using RNA interference to *Bmal1* mRNA. Down-regulation of *Igf1* gene expression was observed by *Bmal1* silencing, whereas the expression of *Inhβa* was significantly increased. During active oscillation of circadian clock, the apoptosis-related genes *Fas* and *Caspase3* remained no significant changes, but they were significantly increased by knockdown of *Bmal1* mRNA. These results indicate that clock-controlled genes are up- or down-regulated in rat UESCs during the stage of decidualization. DNA microarray analysis coupled with RNA interference will be helpful to understand the physiological roles of some oscillating genes in blastocyst implantation and placenta formation.

## Introduction

Circadian rhythms are primarily synchronized with environmental time by the 24-h period of light-dark cycle. In mammals, the master clock in the suprachiasmatic nucleus coordinates the subsidiary oscillators in the majority of peripheral tissues. However, autonomic circadian oscillators are also functional in peripheral tissues. The peripheral circadian oscillators play critical roles in synchronizing local physiology and metabolism to operate in a circadian manner via regulation of the expression of clock-controlled genes (1). These physiological processes mainly include hormonal secretion, gluconeogenesis, lipogenesis, and bile acid homeostasis (2–4). In addition, cellular differentiation may cause the suspension of the cyclic expression of clock genes (5, 6). Recent studies suggest that the circadian system is not only required for proper growth control, but also involved in the circadian regulation of cell proliferation and apoptosis. It has been reported that 2–10% of all mammalian genes are controlled under the circadian clock (1, 7, 8). Most of these genes are involved in organ functions and show tissue-specific expression. Only a small set of the clock-controlled genes are expressed in multiple organs. Among them are genes that encode key regulators of cell cycle progression (9, 10).

Several recent studies have demonstrated that circadian clock genes are rhythmically expressed in the uterus (2, 11–15). In the uterus composed of heterogeneous cell types, ovarian steroids regulate the proliferation and differentiation of uterus endometrial stromal cells (UESCs). In rodents and humans, the UESCs undergo proliferation and differentiation into decidual cells in response to ovarian steroids and blastocyst implantation at the early stage of pregnancy (16–18). This process ultimately results in the formation of the placenta.

Mice lacking the *Clock* gene display abnormal estrus cycles and are infertile (19, 20). Furthermore, implantation fails in *Bmal1* deficient mice, due to impaired steroidogenesis (21), and mutations of *Per1* and *Per2* in mice display reproductive deficits in the middle-aged mutant females (22). More recently, we proved the *Per2* expression is down-regulated in the UESCs during decidualization that influences the expression of vascular endothelial growth factor A (*Vegfa*) gene (23). Deregulation of the circadian clock may attenuate or disrupt expression of the clock-controlled genes and can have a profound influence on organ functions. Studies have demonstrated that the circadian clock function is very important for cell cycle, DNA damage response, and tumor suppression *in vivo* (24–26).

In the present study, to search the clock-controlled genes expressed during the period of implantation, we analyzed the expression of the clock genes and clock-controlled genes in cultured UESCs prepared from pregnant rats at the stage of implantation using DNA microarray technology. We used transgenic rats constructed with mouse *Per2* promoter-destabilized luciferase (*Per2-dLuc*) reporter gene (27) to precisely adjust the time of gene expression. In addition, several genes of significantly expressed genes including growth factor genes and apoptosis-related genes were analyzed using RNA interference (siRNA) to *Bmal1* mRNA to determine whether these were controlled under circadian clockwork.

## Materials and Methods

### Animals

Mouse *Per2* promoter region, assembly by NCBI and the Mouse Genome Sequencing Consortium, was fused to a *dLuc* reporter gene (27). *Per2-dLuc* transgenic rats were generated in accordance with the method described in the patent publication number WO/2002/081682 (Y.S. New Technology Institute, Utsunomiya, Japan). Adult females were mated with fertile males, and 12:00 p.m. on the day of finding spermatozoa in the vaginal smear was designated as day 0.5 of gestation. All the experiments were performed under the control of the Guidelines for Animal Experiments in the Faculty of Medicine, Kyushu University, and Law No. 105 and Notification No. 6 of the Government of Japan.

### Preparation and culture of UESCs

The UESCs were isolated from *Per2-dLuc* transgenic rats on day 4.50 of gestation as reported previously (6, 28, 29). The harvested cells were washed thrice with fresh DMEM/F12, and seeded onto 35 mm collagen-coated dishes at the density of 2 × 10^5^ cells/dish with 2 mL of culture medium (phenol red-free DMEM/F12 supplemented with 10% charcoal-treated FBS and 1× PS). The culture medium was replaced at 15 min after cell seeding to remove epithelial cells. Cells were cultured in a humidified atmosphere of 95% air and 5% CO_2_ at 37°C for 2 days. Then, cells were cultured in serum-free medium supplemented with 1× antibiotic-antimycotic (AA; Nacalai Tesque, Kyoto), 1× Insulin-Transferrin-Selenium (ITS, Life Technologies, Grand Island, NY, USA), 0.1% bovine serum albumin (BSA, Sigma Chemicals), and 100 nM progesterone (P_4_, Sigma Chemicals) for additional 2 days prior to other treatments.

### Real-time monitoring of Per2-dLuc oscillations

The cultured UESCs were synchronized with 100 nM dexamethasone for 2 h in the serum-free medium containing 1× AA. Then, cells were given the serum-free medium DMEM/F12 supplemented with 15 mM HEPES, 0.1 mM luciferin (Wako, Tokyo), 0.1% BSA, 1× AA, and 1× ITS, and subjected to luminescence determination. Luciferase activity was chronologically monitored at 37°C with a Kronos Dio AB-2550 luminometer (ATTO, Tokyo) interfaced to a computer for continuous data acquisition, as described previously (6, 13, 14). The amplitude and period of *Per2-dLuc* oscillations were documented by the single Cosinor method using Timing Series Single 6.3 (Expert Soft Tech., Richelieu, France).

### Microarray analysis

RNA samples isolated from cultured UESCs at 30, 36, 42, and 48 h after dexamethasone synchronization were used for microarray analysis using the Whole Rat Genome Microarray 4 × 44 K Ver3.0 (Agilent Technologies, Santa Clara, CA, USA) representing 30,367 probe sets. The preparation of the samples, microarray hybridizations, and bioinformatics analysis were performed by the Cell Innovator at the Kyushu University (Fukuoka, Japan). Bioinformatics analysis was performed using Agilent Future Extraction software (Agilent Technologies). The data were filtered for signal intensity values (*p*  ≤ 0.05, detectable), which allowed removing very low signal values. After this filtering, 12,252 probe sets remained. Probe sets passing the quality control were then analyzed by ANOVA, and *p*-values of<0.05 were considered significant. The ratio from signal intensity values of four time points was calculated. For the pathway analysis, probe sets were then used for gene ontology analysis using Database for Annotation, Visualization, and Integrated Discovery (DAVID) Bioinformatics Resources (http://david.abcc.ncifcf.gov/) (30). Pathways regulated at *p* < 0.05 were considered significant.

### Bmal1 siRNA transfection

Three sequences targeting the *Bmal1* mRNA and no silencing RNA for rat were purchased from BOVAC Co. (Kurume, Japan). The sequences of RNA oligos used are listed in Table 1. The scrambled RNA for rat was used as no silencing RNA (BOVAC Co.). Both the *Bmal1* siRNA and no silencing RNA were used at final concentrations of 25 nM. The cells were maintained with transfection medium for duration of 12 h (31). Then the medium was replaced with DMEM/F12 supplemented with 1× AA, 1× ITS, 0.1% BSA, and 100 nM P_4_.

**Table 1:** **siRNAsequences targeting*Bmal1* mRNA**.

	Target sequence 5′-3′	siRNA sequence5′-3′
siRNA1	GAAAAGAGGCGUCGGGACA (829–847)	F:GAAAAGAGGCGUCGGGACAdTdT
		R: UGUCCCGACGCCUCUUUUCdTdT
siRNA2	CAGUAAAGGUGGAAGAUAA (1358–1376)	F: CAGUAAAGGUGGAAGAUAAdTdT
		R: UUAUCUUCCACCUUUACUGdTdT
siRNA3	GAGAAAAGAUCACGACUAA (1775–1793)	F: GAGAAAAGAUCACGACUAAdTdT
		R: UUAGUCGUGAUCUUUUCUCdTdT
Non-silencing RNA (control)	–	F: UACUAUUCGACACGCGAAGdTdT
		R: CUUCGCGUGUCGAAUAGUAdTdT

### RNA extraction and RT-qPCR

Cultured cells were harvested at the indicated times, and total RNA was isolated using RNeasy Mini kit (Qiagen), according to the manufacturer’s protocol. RNA samples were treated with RNase-free DNase I (Qiagen). The cDNAs were generated by reverse transcription with random primers using a High Capacity Reverse Transcription kit (Applied Biosystems). One microgram of total RNA were reverse transcribed in 20 μL of mixture using MMLV High Performance Reverse Transcriptase (Epicentre Biotechnologies) and Oligo-dT primer according to the manufacture’s protocol. Primer sets used for real-time PCR were listed in Table 2. PCR was performed with a 1:15 dilution of cDNA samples in Master SYBR Green I mixture (Roche Diagnostics) with specific primers (0.25 μM final of each primer) using Mx3000P Real-time QPCR System (Stratagene). Relative quantification of the mRNA levels was performed using the comparative cycle threshold (ΔCt) method. The ΔCt for each sample was normalized to *Gapdh* and expressed as relative to the control values (23).

**Table 2:** **Primer sequences for the targeted genes in qRT-PCR**.

Gene	Accession No.	Sequence 5′-3′	Amplicon (bp)
*Bmal1*	NM_024362	F: CCGTGGACCAAGGAAGTAGA	97
		R: CTGTGAGCTGTGGGAAGGTT	
*Rev-erb*α	NM_031134	F: ACAGCTGACACCACCCAGATC	102
		R: CATGGGCATAGGTGAAGATTTCT	
*Dbp*	NM_012543	F: GCAAGGAAAGTCCAGGTGCCCG	95
		R: GCGTCTCTCGACCTCTTGGCT	
*Igf1*	NM_178866	F: GTGTCCGCTGCAAGCCTAC	9
		R: CAAGTGTACTTCCTTCTGAGTCTTGG	
*Inhβa*	NM_017128	F: ATGTGCGGATTGCTTGTG	95
		R: CTTCCCGTCTCCATCCA	
*Fas*	NM_139194	F: TGCACCCGGACCCAGAATACCA	133
		R: TGCTGGTTCGTGTGCAAGGCTC	
*Caspase3*	NM_012922	F: GCGGAGCTTGGAACGCGAAGAA	120
		R: ATCGGCAGTGGTGTCGGCGA	
*Gapdh*	NM_017008	F: AACCTGCCAAGTATGATGACATCA	111
		R: ACAACTTCGGCGTCCTCTGTTGGA	

### Statistical analyses

All data are presented as means ± SEM of at least three separate experiments, each performed with triplicate samples. The amplitude of *Per2-dLuc* was documented by Cosinor analysis using Timing Series Single 6.3 (Expert Soft Tech., Richelieu, France). The statistical differences of examined values of target genes in cultured UESCs were determined by Student’s *t*-test using SigmaPlot software (Ver. 11.2; Systat Software, San Jose, CA, USA). Differences were considered significant at *p* < 0.05 or less.

## Results

### DNA microarray analysis of oscillating genes expressed in the rat UESCs after dexamethasone synchronization

**Figure 1:**
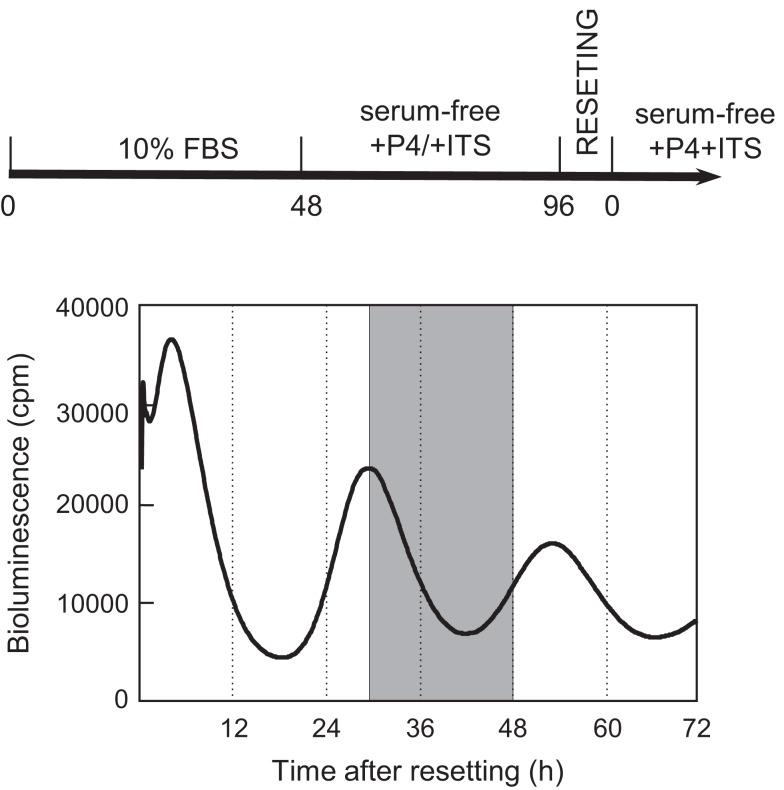
**Isolation protocols of total RNA samples from cultured uterus endometrial stromal cells after dexamethasone synchronization**. According to the second to the third phases of the *Per2-dLuc* oscillation, total RNA samples (*n* = 3) individually isolated from cultured UESCs at 30, 36, 42, and 48 h (*shadow area*) after dexamethasone synchronization were used for microarray analysis.

During monitoring of *Per2-dLuc* activity oscillation, RNA samples were prepared at 30, 36, 42, and 48 h after dexamethasone synchronization and global gene expression patterns were determined using DNA microarray technology (Figure 1). The analysis revealed 7,235 significantly altered genes in the UESCs. The increased expression (357 genes) and decreased expression (202 genes) of genes showing with fold change were obtained from 7,235 significantly altered genes at four time points during oscillation (Table 3). The majority of fold-changed genes were identified in both RNA samples 30 versus 48 h and 36 versus 48 h, including growth factors, transcription factors, receptors, channels, and enzymes (Table 4). For example, the expression of growth factors such as *Inhβb, Gdf10*, and *Gdf15* were increased during the interval, whereas the expression of *Igf1* was decreased. To place the microarray results in the cellular context, we performed the biological pathway analysis using the DAVID online annotation software. This analysis revealed that genes were involved in cell cycle, glutathione metabolism, MAPK signaling pathway, fatty acid metabolism, ubiquitin mediated proteolysis, focal adhesion, PPAR signaling pathway, and so on (Table 5). It was identified that the expression of 38 cell cycle related genes were significantly altered during the second to third phase of *Per2-dLuc* activity oscillation (*p* < 0.00001).

**Table 3:** **Number of altered genes with fold change in the rat UESCs as revealed by microarray analysis**.

Sampling time (h)	Number of genes			
	30	36	42	48
30	–	9	17	179
36	8	–	14	90
42	30	27	–	48
48	90	37	10	–

*Red: number of up-regulated genes*.

*Blue: number of down-regulated genes*.

**Table 4:** **Representative genes altered with fold change as revealed by DNA microarray**.

Category	Accession No.	Representative genes (gene symbols)	Direction of change*
Growth factors	NM_080771	Inhibinβ-B (*Inhβb*)	Up
	NM_024375	Growth differentiation factor 10 (*Gdf10*)	Up
	NM_019216	Growth differentiation factor 15 (*Gdf15*)	Up
	NM_012561	Follistatin (*Fst*)	Down
	NM_178866	Insulin-like growth factor 1 (*Igf1*)	Down
Transcription factors	NM_001108393	Zinc finger, MIZ-type containing 1 (*Zmiz1*)	Down
	NM_012543	D site of albumin promoter binding protein (*Dbp*)	Up
	NM_001011998	F-box protein 9 (*Fbxo9*)	Down
Receptors	NM_001025680	G-protein-coupled receptor 4 (*Gpr4*)	Up
	NM_133306	Oxidized low density lipoprotein receptor 1 (*Olr1*)	Up
Channels	NM_012778	Aquaporin-1 (*Aqp1*)	Up
G-protein signaling	NM_053453	Regulator of G-protein signaling 2 (*Rgs2*)	Down
	NM_019341	Regulator of G-protein signaling 5 (*Rgs5*)	Down
Enzymes	NM_133530	Matrix metallopeptidase 13 (*Mmp13*)	Up
	NM_001108344	Ubiquitin-conjugating enzyme E2T(*Ube2t*)	Up
	NM_001029904	Ribonuclease1 (*Rnase1*)	Up
	NM_017000	Quinone 1 (*Nqo1*)	Up

*Compared at 30 versus 48 h or 36 versus 48 h.

**Table 5:** **Pathway analysis of DNA microarray using DAVID online annotation software**.

Pathways (gene No.)	Representative genes contained within each pathway	*p*-Value
Cell cycle (38)	*Cdc23, Cdc45, Bub1, Bub3, Ccna2, Ccnb1, Ccnd3, Cdkn1b, Cdkn1c, Gadd45a, Plk1, Bmyc, Mdm2*	<0.00001
Glutathione metabolism (19)	*Gsta3, Gsta5, Gstk1, Gstm1, Gsto1, Gstm5, Gstt2, Idh1, Mgst1, Mgst2, Srm*	<0.0001
MAPK signaling (55)	*Mknk2, Rasa1, Chp, Cacna2d1, Cacna1i, Cacna1g, Cacna1i, Cacna1c, Dusp5, Dusp6, Gadd45g, Gnb4, Map3k7, Mapk8ip1, Map3k6, Map4k4, Pla2g6, Ppm1a, Ppm1b*	0.00411
Fatty acid metabolism (14)	*Adh1, Aldh3a2, Aldh9a1*	0.00452
Ubiquitin mediated proteolysis (30)	*Cul5, Pias4, Ttc27, Tceb2, Ube3c, Ube2j1, Ube2b, Ube2cbp, Ube2d3, Ube2k, Ube2s, Ube2z, Uba1, Ube4a*	0.00559
Focal adhesion (42)	*Col1a1, Col1a2, Col5a1, Col6a1, Itfg1, Itgb1bp1, Itga11, Itgb5, Lamb2*	0.00631
PPAR signaling (19)	*Fads2, Lpl, Me1, Olr1, Pltp, Scd, Scd1*	0.00968
mTOR signaling (15)	*Ddit4l, Cab39l, Eif4ebp2, Eif4ebp1, Rptor, Rps6ka1, Pdpk1*	0.0148
Neurotrophin signaling (28)	*Irak3, Ngf, Ngfrap1, Plcg1, Psen1, Pdk1, Nras, Rac1, Raf1*	0.0189
Endocytosis (40)	*Arfgap1, Atg2a, Asap1, Acap2, Ehd4, Git2, Rt1aa, Rab22a, Sh3glb1, Sh3kbp1, Dab2, Lrp11*	0.0272
Gap junction (19)	*Csnk1d, Gnai2, Tjp1, Tubb2a, Tubb3, Tubb4a, Gnas, Lpar1*	0.0356
SNARE interactions in vesicular transport (11)	*Snap23, Stx12, Stx1a, Vamp2, Vamp3, Vamp4, Vamp7, Vamp8*	0.0375

### Functional molecular machinery of a circadian clock exists in the rat UESCs after dexamethasone synchronization

**Figure 2:**
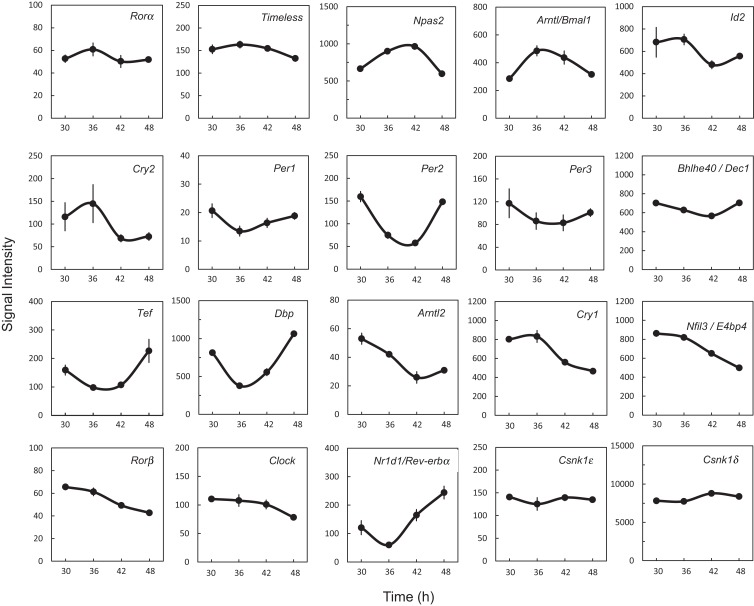
**Expression profiles of clock genes and protein kinase genes in the uterus endometrial stromal cells of pregnant rats after dexamethasone synchronization**. Each value represents the means ± SEM (*n* = 3) of signal intensity from the microarray results.

**Figure 3:**
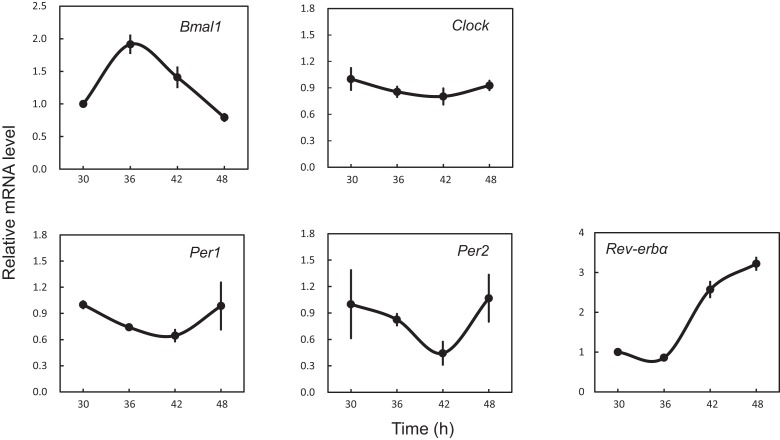
**Expression profiles of core clock genes in the uterus endometrial stromal cells after dexamethasone synchronization as revealed by RT-qPCR**. According to the *Per2-dLuc* oscillation as illustrated in Figure 1, total RNA samples were collected at the indicated times after synchronization. qRT-PCR analyses of transcript levels were performed using their specific primers. *Gapdh* was used as an internal control. Each value represents the mean ± SEM (*n* = 3).

In order to determine whether 18 clock genes displayed diurnal rhythmic expression, we performed gene clustering on the microarray results. The gene expression profiles were mainly divided into four groups: the first group was *Ror*α, *Timeless, Npas2, Bmal1* (*Arntl*), *Id2*, and *Cry2*; the second group *Per1, Per2, Per3, Dec1* (*Bhlhe40*), *Tef*, and *Dbp*; the third group *Bmal2* (*Arntl2*), *Cry1, E4bp4* (*Nfil3*), *Ror*β, and *Clock*; the fourth group *Rev-erb*α (*Nr1d1*) (Figure S1 in Supplementary Material). The *Per2* intensity profile was consistent with *Per2* circadian oscillation (Figure 2). Profiles of *Bmal1* and *Rev-erb*α expression were obviously anti-phase in the UESCs, because Bmal1 promotes *Rev-erb*α transcription with Clock and in turn Rev-erbα inhibits *Bmal1* transcription through its binding to the RORE in the promoter region. The *Per2* and *Dbp* expression profiles were also out-of-phase with the *Bmal1* rhythm. The protein kinase genes such as *Csnk1ε* and *Csnk1*δ also displayed the weak rhythmic expression (Figure 2). The expression profiles of core clock genes such as *Bmal1, Clock, Per1, Per2*, and *Rev-erb*α were further confirmed by RT-qPCR. As the results are shown in Figure 3, their profiles were very similar to the microarray results. The Cosinor analysis method was used to determine the rhythmic expression of examined genes. *Bmal1, Per1, Per2*, and *Rev-erb*α displayed rhythmic expression (*p* < 0.05).

### Significant alterations of genes related to either implantation or placenta formation

In order to determine whether genes involved in implantation or placenta formation displayed significant alteration, we performed gene clustering on the microarray results. As the gene clustering is shown in Figure S2 in Supplementary Material, 11 genes of 33 implantation-related genes displayed significant alterations (*p* < 0.05). Of 11 significantly altered genes, three genes such as *Bsg, Pcsk5*, and *Klf9* were up-regulated, whereas *Tgfβr2, Ppar*δ, *Ptgs2, Grn*, and *Fkbp4* were significantly down-regulated.

The results of gene clustering for placenta formation are shown in Figure S3 in Supplementary Material. Twenty-four genes of 107 placenta formation-related genes displayed significant alterations (*p* < 0.05). Of 24 significantly altered genes, nine genes *Txnrd1, Pgf, Pkd2, Hif1a, Ccnf, Plac9, Prdx3, Adm*, and *Pbrm1* were up-regulated, whereas *Plac8, Prdm1, Rspo3, Tmed2, Dcn, Epas1*, and *Stk3* were significantly down-regulated. These results indicate that these genes involved in implantation and placenta formation may be controlled under circadian clock.

### Up-regulation and down-regulation of genes related to cell growth and apoptosis

**Figure 4:**
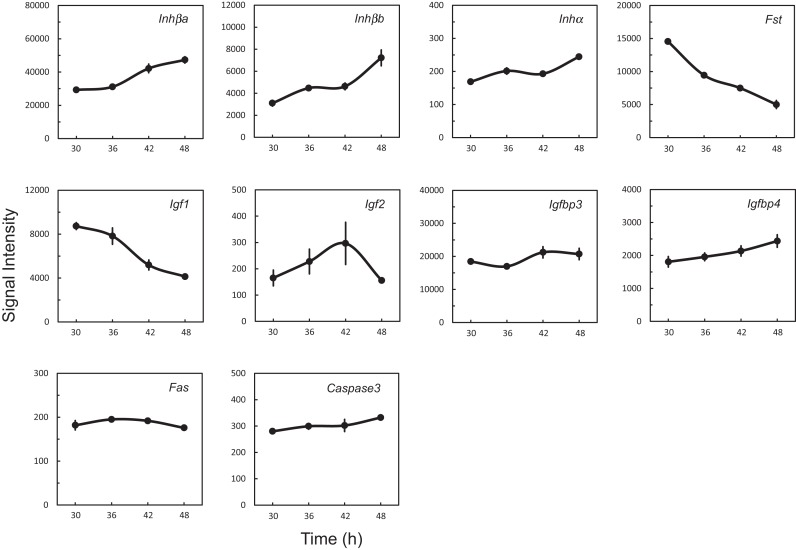
**Characteristic expression profiles of genes coding growth factors, their binding proteins, and apoptosis-related proteins**. Each value represents the means ± SEM (*n* = 3) of signal intensity from the microarray results.

Of fold-changed genes (Table 4), we focused on *Inhβb* and *Igf1* that were regulated with opposite directionality. Both *Inhβa* and *Inhβb* were high in signal intensity, whereas *Inha* was low, indicating that activin, but not inhibin, is highly produced in the UESCs. The *Fst* gene coding the activin-binding protein follistatin was down-regulated. In contrast to *Inhβa* and *Inhβb, Igf1* was down-regulated, whereas its binding proteins *Igfbp3* and *Ifgbp4* were rather increased (Figure 4). Consequently, the activities of activin and IGF1 may be differentially regulated during circadian rhythms. During active oscillation of circadian clock, however, the apoptosis-related genes *Fas* and *Caspase3* remained no significant changes (Figure 4).

### Effect of Bmal1 knockdown on the expression of genes related to cell growth and apoptosis in the rat UESCs

**Figure 5:**
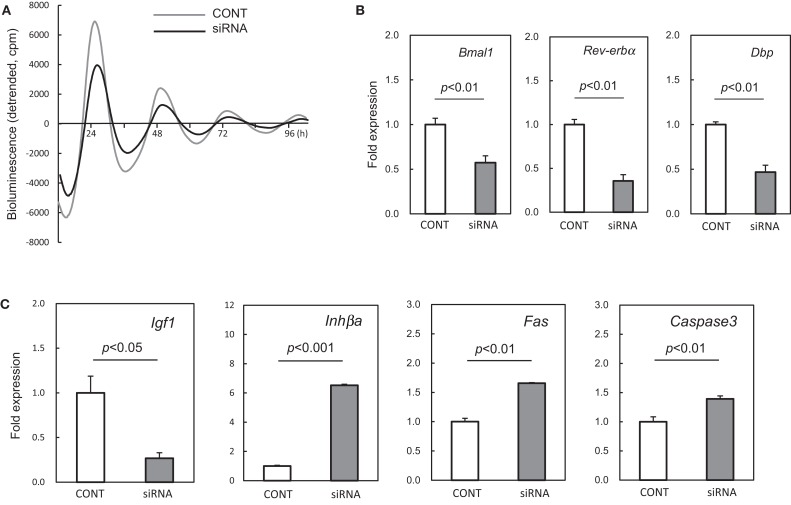
**Effect of *Bmal1* siRNA treatment on expression of putative clock-controlled genes in cultured uterus endometrial stromal cells**. **(A)** The UESCs were treated with *Bmal1* siRNA (siRNA) or no silencing RNA (CONT) according to the indicated protocols. The cells were then synchronized with dexamethasone for bioluminescence determination (time: 0 h), and then monitored *Per2-dLuc* oscillations. **(B)** RNA samples were collected 39 h after synchronization, and RT-qPCR analyses of transcript levels were performed using their specific primers. **(C)** Transcript levels of *Igf1, Inhβa, Fas*, and *Caspase3* were measured in the UESCs treated with siRNA or no silencing RNA. Each value represents the means ± SEM (*n* = 3).

In order to suppress the cellular clock in the UESCs, *Per2-dLuc* oscillations were investigated using *Bmal1* siRNA (siRNA) or no silencing RNA (CONT). The UESCs transfected with either RNA displayed several *Per2-dLuc* oscillations. A decline of *Per2-dLuc* bioluminescence oscillation and significantly decreased amplitude (*p* < 0.01, versus CONT) were observed in *Bmal1* siRNA treated cells (Figure 5A). There was not a significant effect of *Bmal1* siRNA treatment on the peak time of the first phase and the cycle time (data not shown). The transcript levels of core clock genes were estimated at the peak time of the first phase. The results are shown in Figure 5B. *Bmal1* mRNA expression was inhibited after transfection with *Bmal1* siRNA, the *Bmal1* transcript level being reduced by 60% compared with that in the CONT group. *Rev-erb*α and *Dbp* are regarded as core circadian oscillator genes that fall under the regulation of Bmal1-Clock heterodimer through E-box elements located in their promoter regions. Thus *Rev-erb*α and *Dbp* were significantly down-regulated in *Bmal1* siRNA cells.

In addition, we observed that the mRNA expression of several uterus genes (*Igf1, Inhβa, Fas*, and *Caspase3*) was differentially affected by the *Bmal1* siRNA treatment. The expression of *Igf1* gene was significantly down-regulated by *Bmal1* silencing (*p* < 0.05), whereas the *Inhβa* was up-regulated approximately sevenfold (*p* < 0.001) (Figure 5C). Although *Fas*, and *Caspase3* did not exhibit cellular circadian rhythms as described above, these genes were significantly up-regulated by *Bmal1* silencing (*p* < 0.01).

## Discussion

The present analysis of circadian genes regulated by clockwork system significantly contributes to our understanding of the relationship of cellular circadian clock with implantation and decidualization. Our present study on cultured UESCs of pregnant rats is the first to analyze the global gene expression profiles using DNA microarray technology. We found the specific expression profiles of 18 clock genes and two protein kinase genes. *Bmal1* (*Arntl*) and *Rev-erb*α (*Nr1d1*) displayed typically anti-phase expression patterns, showing that the proper range of clock gene oscillation was successfully covered. We identified 7,235 significantly altered genes in the UESCs and 559-fold-changed genes with up or down directionality. The biological pathway analysis using the DAVID online annotation software revealed that many genes involved in cell cycle (38 genes), glutathione metabolism (19 genes), MAPK signaling pathway (55 genes), and so on were significantly expressed with circadian regulation. In addition, some genes involved in implantation or placenta formation displayed significant alteration with up or down directionality: 11 genes involved in implantation and 24 genes involved in placenta formation.

Many genes were listed as the up-regulated genes compared to the RNA sample at 30 h, including *Inhβb, Gdf10* (*BMP-3*), *Gdf15* (*MIC-1*), *Dbp, Gpr4*, and *Olr1*. Of 559-fold-changed genes, *Gdf10*, a member of TGFβ superfamily related to BMP-3 (32), is required for normal decidual development during the post-implantation period (33). *Gdf15* (MIC-1) is expressed in the decidual stromal cells and trophoblasts (34). GPR4, a proton-sensing G-protein-coupled receptor, is also expressed in human umbilical vein endothelial cells (35). GPR4 signaling regulates endothelial cell adhesion through the cAMP pathway. It is known that the oxidized low density lipoprotein receptor 1 (*Olr1*) regulates growth of a variety of cells and is important in inflammation, oxidative stress, and tissue remodeling (36). However, the expression and regulation of *Olr1* remain unclear in the uterus. Aquaporin-1 (*Aqp1*) is proposed as a mediator of estrogen-induced angiogenesis in breast cancer and endometrial cancer (37). The expression of *Aqp1* was reported in the pregnant rat uterus (38, 39). Although more than 10 *Aqp1* have been identified up to date, only *Aqp1* was expressed in the cultured UESCs.

In contrast, the down-regulated representative genes with fold change were also listed, including *Fst, Igf1, Zmiz1, Fbxo9, Rgs2, Rgs5, Mt1a*, and *Mt2A*. IGF family genes including *Igf1* are differentially expressed throughout gestation, and especially the IGF system contributes phenotypic and functional changes of myometrium smooth muscle cells (40). The overexpression of *Zmiz1*, recently identified as a candidate oncogene, was reported in human breast, ovarian, and colon cancers (41). *Zmiz1* was also reported to function in regulating the activity of the androgen receptor as a co-activator (42). However, the expression and function of *Zmiz1* are unclear in the uterus. *Fbxo9* was reported to contribute survival of myeloma cells (43). *Rgs2* and *Rgs5*, regulators of G-protein signaling proteins, are abundantly expressed in pregnant human myometrium (44). *Rgs2* was reported to up-regulate in response to stress (45). The expression of *Mt1a* and *Mt2A*, typical metal response parameters, was reported in the proliferative phase of endometrium (46).

Of up-regulated and down-regulated genes, we focused on the expression of *Inhβb, Inhβa*, and *Igf1* that displayed fold change with up or down directionality. Both the *Inhβa* and *Inhβb* genes showed high signal intensities of DNA microarray, whereas *Inh*α was low. This result indicates the expression of activin. The binding protein of activin, follistatin (*Fst*), was down-regulated, indicating the relative action of activin is increased. Expression of activin A was increased with reduced stromal cell mitosis, tissue growth, and mitogenic signaling in the decidual endometrium (47). We analyzed using *Bmal1* siRNA whether the expression of *Inhβa* and *Igf1* was controlled under circadian clock. Transfection of *Bmal1* siRNA into the UESCs induced a significant decline of *Per2-dLuc* bioluminescence oscillation. However, the transfection did not alter other parameters in the oscillation. The core clock genes *Rev-erb*α and *Dbp* as well as *Bmal1* were down-regulated. The expression of *Igf1* was significantly decreased after *Bmal1* silencing, suggesting that *Igf1* is positively regulated by circadian clock. Conversely, the expression of *Inhβa* was enhanced approximately sevenfold after *Bmal1* silencing, suggesting that *Inhβa* is negatively regulated by circadian clock. Although both *Inhβa* and *Igf1* are clock-controlled genes, thus, the two gene are differentially regulated. Rev-erbα is a critical component of circadian clock (48). It acts as a domain clock-controlled gene under the regulation of Bmal1-Clock heterodimer; in turn it has been shown to suppress *Bmal1* transcription through binding to RORα response elements (RORE) presenting in *Bmal1* promoters (49). Recent studies have shown that Rev-erbα is a transcriptional silencer (49, 50). However, Rev-erbα increases the transcription of *Star* by binding to its agonist heme (51).

In the uterus endometrial cells of pregnant rats, the early proliferative phase is characterized by tissue remodeling, angiogenesis, and modulation of inflammation; the mid-proliferative phase is characterized not only by proliferation but also marks the onset of expression of genes required for endometrial receptivity. Thus, cell growth and apoptosis support the process of tissue remodeling and implantation. The death receptor Fas/ligand system is a key regulator of apoptotic cell death and irregularity of this signaling pathway has been shown to participate in immune-mediated β-cell apoptosis (52, 53). It is also known that the expression level of the *Fas* gene is down-regulated in a variety of malignancies including malignant melanoma, adenocarcinoma, and squamous cell carcinoma (54, 55). In the cultured UESCs, however, the expression of *Fas* and *Caspase3* displayed no or small changes. Interestingly, these genes were significantly up-regulated after *Bmal1* silencing, similar to *Inhβa*, suggesting that these apoptosis-related genes are suppressed during active circadian rhythms of clock genes. It is possible that dysfunction of the circadian clockwork during the stage of decidualization is necessary to increase or decrease expression of clock-controlled genes for formation of the placenta.

## Conflict of Interest Statement

The authors declare that the research was conducted in the absence of any commercial or financial relationships that could be construed as a potential conflict of interest.

## Supplementary Material

The Supplementary Material for this article can be found online at http://www.frontiersin.org/Systems_and_Translational_Endocrinology/10.3389/fendo.2013.00082/abstract

Supplementary Figure S1 **Clustering of clock genes on the microarray results**. The expression profiles of clock genes (*closed circle*) were divided into four groups (1−4). *Red*, relatively high expression; *green*, relatively low expression.

Supplementary Figure S2 **Clustering of implantation-related genes on the microarray results**. Genes showing with significant alterations (*p* < 0.05) are listed. *Red*, relatively high expression; *green*, relatively low expression.

Supplementary Figure S3 **Clustering of placenta formation-related genes on the microarray results**. Genes showing with significant alterations (*p* < 0.05) are listed. *Red*, relatively high expression; *green*, relatively low expression.
