# Supplementary figures and images for: Profiling of Circadian Genes Expressed in the Uterus Endometrial Stromal Cells of Pregnant Rats as Revealed by DNA Microarray Coupled with RNA Interference
Source: Front Endocrinol (Lausanne). 2013 Jul 8;4:82. doi: 10.3389/fendo.2013.00082 (PMC3703733; doi:10.3389/fendo.2013.00082)

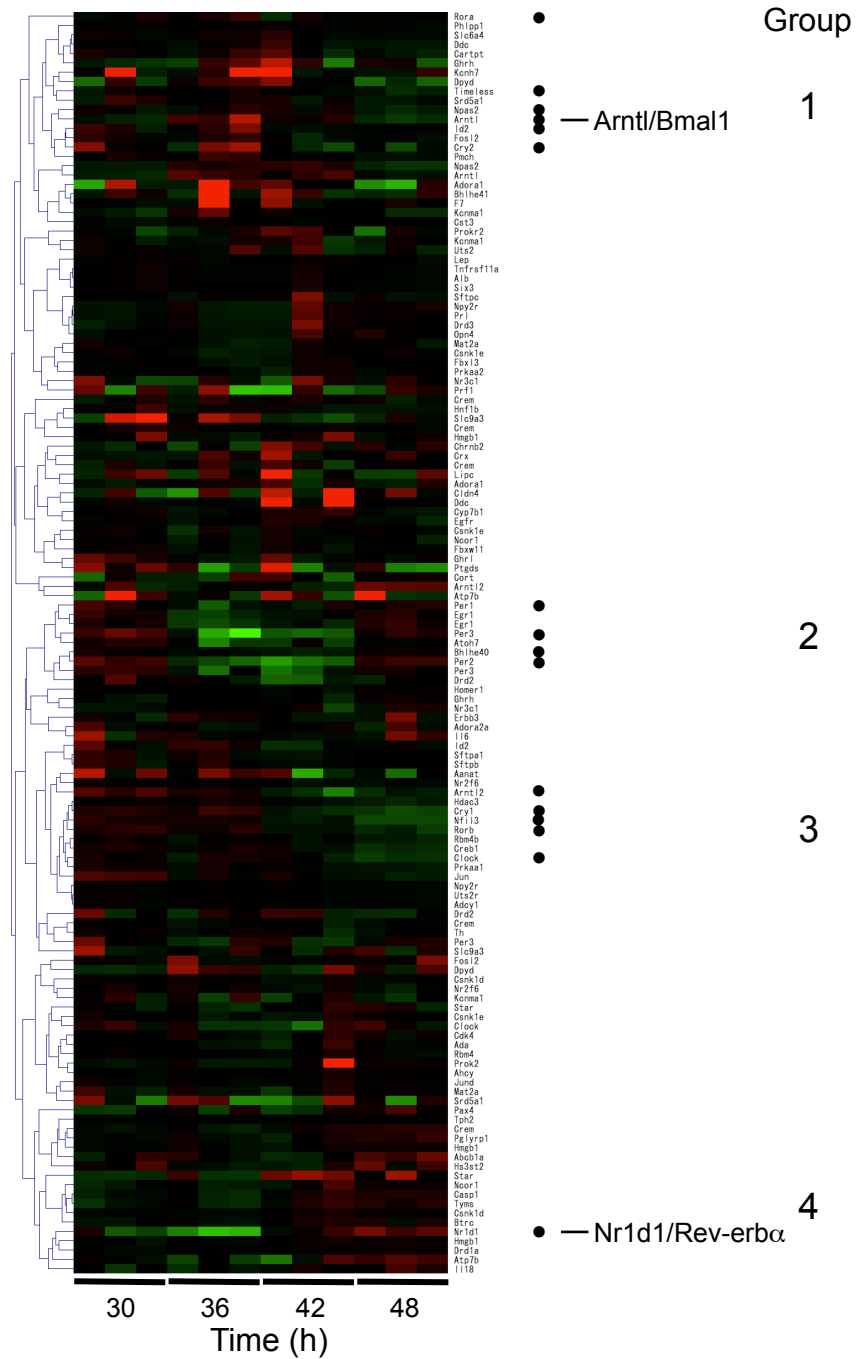

Supplement: Supplementary Figure S1 — Clustering of clock genes on the microarray results. The expression profiles of clock genes (closed circle) were divided into four groups (1−4). Red, relatively high expression; green, relatively low expression. [file 51785_Hattori_DataSheet1.PDF]

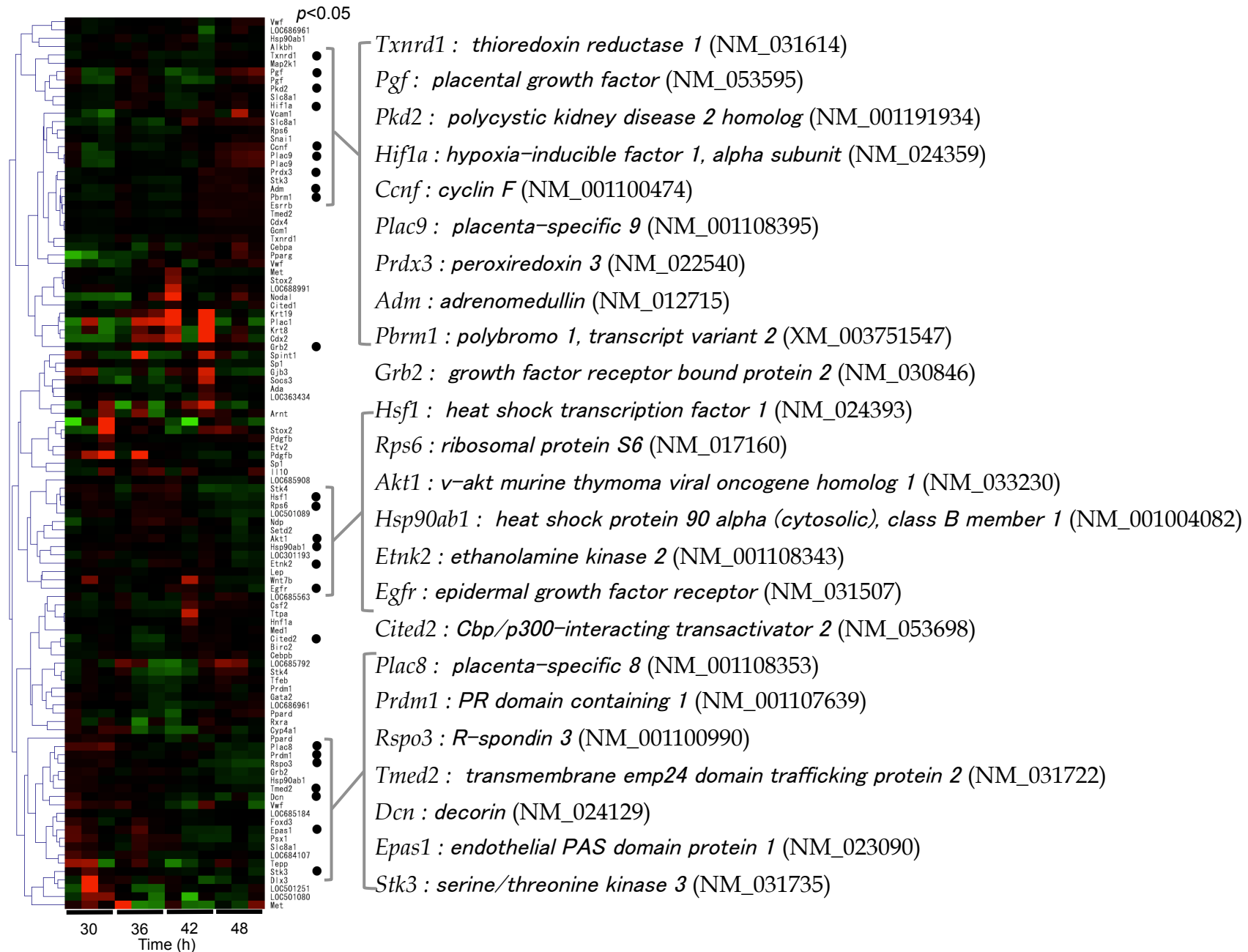

Supplement: Supplementary Figure S3 — Clustering of placenta formation-related genes on the microarray results. Genes showing with significant alterations (p < 0.05) are listed. Red, relatively high expression; green, relatively low expression. [file 51785_Hattori_DataSheet3.PDF]
