# Supplementary material for: Profiling of Circadian Genes Expressed in the Uterus Endometrial Stromal Cells of Pregnant Rats as Revealed by DNA Microarray Coupled with RNA Interference
Source: Front Endocrinol (Lausanne). 2013 Jul 8;4:82. doi: 10.3389/fendo.2013.00082 (PMC3703733; doi:10.3389/fendo.2013.00082)
Supplement: Supplementary Figure S2 — Clustering of implantation-related genes on the microarray results. Genes showing with significant alterations (p < 0.05) are listed. Red, relatively high expression; green, relatively low expression. [file 51785_Hattori_DataSheet2.PDF]

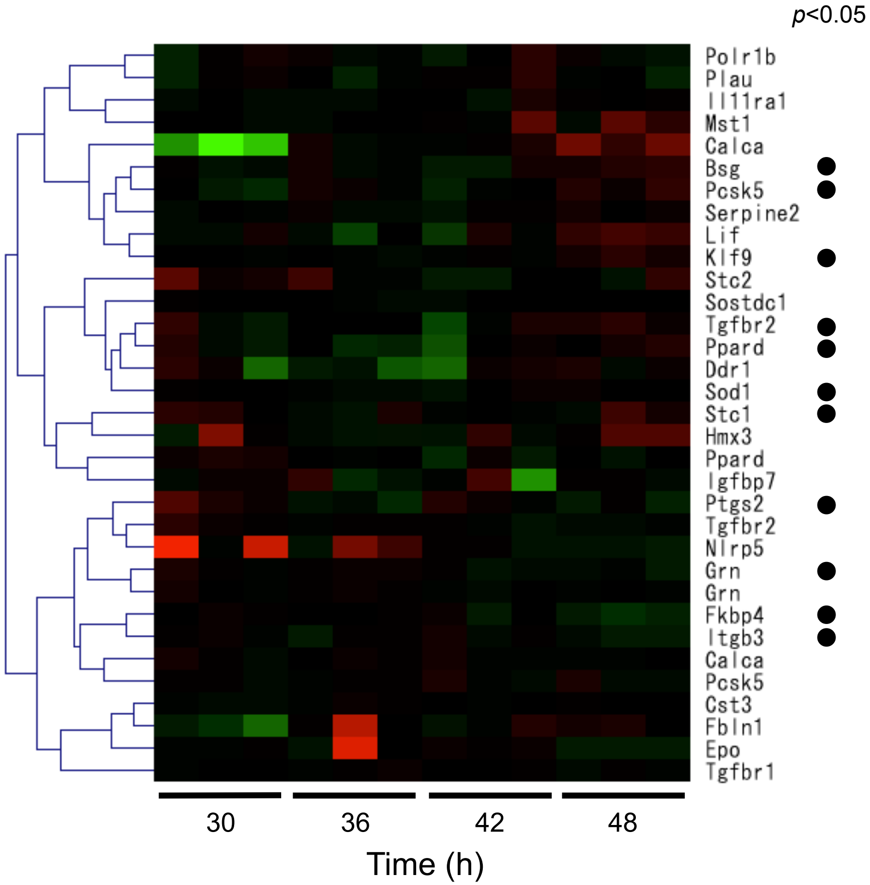

Bsg : *basigin* (NM_001109882)

Pcsk5 : *Proprotein convertase subtilisin/kexin type 5* (NM_053823)

Klf9 : *Kruppel-like factor 9* (NM_057211)

Tgfbr2 : *transforming growth factor, beta receptor II* (NM_031132)

Ppard : *peroxisome proliferator-activated receptor delta* (NM_013141)

Sod1 : *superoxide dismutase* 1 (NM_017050)

Stc1 : *stanniocalcin 1* (NM_031123)

Ptgs2 : *prostaglandin-endoperoxide synthase 2* (NM_017232)

Grn : *granulin* (NM_017113)

Fkbp4 : *FK506 binding protein 4* (NM_001191863)

Itgb3 : *integrin, beta 3* (NM_153720)
